# Closing the gaps in defining and conceptualising acceptability of healthcare: a qualitative thematic content analysis

**DOI:** 10.4314/ahs.v22i3.75

**Published:** 2022-09

**Authors:** Joy Blaise Bucyibaruta, Doriccah Peu, Lesley Bamford, Annatjie van der Wath

**Affiliations:** 1 School of Health Systems and Public Health, University of Pretoria; 2 Department of Homeopathy, University of Johannesburg; 3 Department of Nursing Science, University of Pretoria; 4 National Department of Health, South Africa

**Keywords:** Defining and conceptualising acceptability, healthcare, content analysis

## Abstract

**Introduction:**

Despite the importance of healthcare acceptability, the public health community has yet to agree on its explicit definition and conceptual framework. We explored different definitions and conceptual frameworks of healthcare acceptability, and identified commonalities in order to develop an integrated definition and conceptual framework of healthcare acceptability.

**Materials and Methods:**

We applied qualitative thematic content analysis on research articles that attempted to define healthcare acceptability. We searched online databases and purposefully selected relevant articles that we imported into ATLAS.ti 8.4 for deductive and inductive analysis which continued until there were no new information emerging from selected documents (data saturation).

**Results:**

Our analysis of the literature affirmed that healthcare acceptability remains poorly defined; limiting its application in public health. We proposed a practical definition attempting to fill identified gaps. We defined acceptability as a “multi-construct concept describing the nonlinear cumulative combination in parts or in whole of the fit between the expected and experienced healthcare from the patient, provider or healthcare systems and policy perspectives in a given context.”

**Practice Implications:**

We presented and described a workable definition and framework of healthcare acceptability that can be applied to different actors including patients, healthcare providers, researchers, managers or policy makers.

## Introduction

Acceptability of healthcare is gaining momentum in the literature and is evolving as an emerging discipline of public health^1^. Healthcare acceptability has become a vital and strategic factor in designing, implementing, monitoring and assessing healthcare systems and policy interventions^2^. Despite the importance of healthcare acceptability, the public health community is still lacking a comprehensive definition and conceptual framework of acceptability.

Acceptability of healthcare is a complex and many-sided concept describing appropriateness of healthcare^1^–^3^. The concept reflects interactions amongst patients, healthcare providers as well as healthcare system managers and policymakers^1^, ^3^. Acceptability should thus be considered in the context of patients, healthcare provider or healthcare system managers and policymakers. Although acceptability can be approached as a stand-alone concept, it is widely recognised as one of the dimensions of access to healthcare^4^ that encompasses the social and cultural factors that influence access to healthcare^3^, ^4^. Acceptability is characterised by terms conveying beliefs, perceptions, attitudes and experiences, and how these factors influence the use of healthcare services^3^–^5^. Healthcare users are personally influenced by certain feelings such as privacy, confidentiality, trust, understanding and respect^1^–^5^. These terms often have broad meaning and overlapping values5. Many researchers have argued that these terms should be categorised under specific constructs of acceptability based on the best-fit theory^1^, ^3^, ^5^.

Given the broad meaning of terms associated with human interactions and perceptions, the concept of acceptability in healthcare remains poorly defined^1^, ^2^. Existing literature also reveals a poorly defined conceptual framework^1^, ^2^, ^6^. The lack of clarity makes it difficult to operationalize the concept of acceptability especially from healthcare systems and policy point of view. There is also little research investigating acceptability from healthcare providers' perspectives, indeed most publications approach acceptability from patients' perspectives^1^, ^2^, ^5^. Thus, in an effort to create a workable definition and framework of healthcare acceptability for the public healthcare community, we explored existing definitions and conceptual frameworks of healthcare acceptability. Specifically, we (1) explored and described the complexity of acceptability within the context of access to healthcare; (2) re-examined and clarified the context and semantic domains of acceptability of healthcare to inform its definition and (3) reviewed and elucidated the conceptual framework of acceptability of healthcare and its interpretation.

## Materials and Methods

We conducted a qualitative thematic content analysis on identified articles on acceptability of healthcare^7^. In this case, we searched MEDLINE/PubMed, Cochrane Library and Google Scholar databases for relevant papers, using acceptability of healthcare as keywords. We refined the database search by adding terms such as concept, conceptualisation, construct, define and framework of acceptability in various combinations as our search strategy. Using a snowball strategy, we checked the reference lists of retrieved papers to identify additional documents. We included only full-text English documents that were freely available or accessed via the University of Pretoria Library Portal. We did not consider the date of publication and the quality of identified articles as exclusion criteria.

Following retrieval of purposively selected research articles in line with the developed search strategy, we imported them into ATLAS.ti 8.4 for analysis. We deductively and inductively coded and categorised the themes related to definitions and frameworks of healthcare acceptability^7^. We combined both deductive and inductive coding approaches to get more in-depth from collected data on acceptability^7^; a complex concept that remains poorly defined and conceptualised^1^, ^2^. Based on our knowledge on the topic, we applied a deductive approach to predetermine a set of codes and subcodes used to categorise corresponding themes and subthemes during data analysis. Mindful of the lack of common definition and shared conceptual framework of acceptability, we also applied an inductive approach to develop new codes and subcodes from immerging relevant themes and subthemes in data analysis. Therefore, it was appropriate to combine both deductive and inductive coding approaches to increase transparency and reflexivity for the findings in line with this paper's objectives^7^ We applied a stakeholder analysis to identify networks of actors that have a vested interest in a coherent definition and framework of healthcare acceptability^8^. We also used the actor-networks theory to make sense of identified interconnections and to decide which actor had the largest vested interest in healthcare acceptability^9^.

To ensure the validity, the researchers discussed the preliminary coding system developed by the principal investigator; they revised it until a final coding system was adopted10. The researchers also assessed the intra-coding reliability for the first ten coded documents and there was a perfect agreement (100%) in length and location for the relevant codes^10^. We conducted analysis until no new information emerged anymore from selected documents; a phenomenon called data saturation^7^. This phenomenon was reached after data analysis from 174 out of 500 retrieved articles. The Faculty of Health Sciences Research Ethics committee has approved the research project involving this article (Ethics reference No: 547/2019).

## Results

The results of this study were based on a selection of 174 published articles after which the analysis did not show any new information (data saturation). There were six main themes emerging from the documents analysed including: (1) acceptability within the context of access to healthcare, (2) complexity of acceptability,, (3) context of acceptability, (4) semantic domains of acceptability, (5) definition of acceptability and (6) conceptual framework of acceptability.

### Acceptability within the context of access to healthcare

The concept of access to healthcare was introduced in healthcare literature around the early 1970s^11^. Different researchers have recognised the complex nature of access to healthcare. Many authors have contemplated that the definition of access to healthcare should go beyond simply travelling to the healthcare facility. In fact, some authors have theorised access to healthcare as a “functional relationship” between people and medical facilities providing healthcare^11^, ^12^. Acceptability of healthcare was also thought to encompass enablers and barriers for the people benefiting from available healthcare^11^, ^12^. Though some authors initially understood access to healthcare as a complex concept, specific dimension(s) were not ascribed to this concept in 1971. It took about five years to recognise affordability or financial accessibility and availability o physical accessibility as two dimensions of access to healthcare^13^. Since then, various authors have attempted to improve the definition and conceptual framework of access to healthcare with a variety of definitions and frameworks^14^, ^15^.

It was beyond the scope of this paper to discuss the definition of access to healthcare and different conceptual frameworks, but it was important to note that many authors widely recognised acceptability as one of the dimensions of access^4^, ^14^, ^16^, ^17^.

### Complexity of acceptability

Some authors have referred to acceptability of healthcare as a unitary construct2 without clearly integrating the different elements or constructs of acceptability1. Other authors have used terms such as acceptance, satisfaction, feasibility, enjoyment and uptake as proxies for acceptability^6^. There has been a growing evidence in support of these proxy terms to be differentiated from the concept of acceptability^2^, ^6^.

Initial publication proposed acceptability as a complex concept describing the best fit between the healthcare expectations of the patient and the healthcare system^12^ Subsequently, many researchers have expanded on the original definition. Acceptability was later referred to as socio-cultural accessibility^2^, ^3^, ^6^ ,^18^ Gilson proposed three elements of acceptability namely patient-provider, patient-health service organisation and patient-community interactions3. More recently Sekhon and colleagues defined acceptability as “a multi-faceted construct that reflects the extent to which people delivering or receiving a healthcare intervention consider it to be appropriate, based on anticipated or experienced cognitive and emotional responses to the intervention”^2^.

### Context of acceptability

Most of the articles we analysed emphasised that acceptability of healthcare could only be interpreted effectively if the context was considered1–6. However, the context of acceptability was not clearly described in most of the analysed studies. The analysed articles showed that the context of acceptability goes beyond the setting and population, embracing the content, scope and focus of acceptability. Nevertheless, most of the studies grappled to define the scope and focus of healthcare acceptability. Researchers used one of two theories to define the scope of acceptability of healthcare; acceptability was either referred to as a unitary or a multi-construct concept^2^. In terms of stakeholders' analysis point of view, many researchers approached acceptability of healthcare from patients' perspectives^1^, ^2^, ^5^. Some articles mentioned acceptability from healthcare providers' point of view but did not clearly explain how to apply their definition in practice^2^. Few articles considered healthcare acceptabiity from a healthcare systems and policy (decision makers or managers) perspective.

### Semantic domains of acceptability

The concept of acceptability of healthcare is broad5 and encompasses components with overlapping meanings^2^. Many researchers have suggested using the best-fit theory to assign components into the most appropriate constructs1. This means that one component only should be used to describe no more than one construct. The components used to describe the constructs of acceptability should thus remain mutually exclusive. Some authors have described acceptability as a multi-levelled or multi-layered complex concept^5^. However, much confusion surrounding healthcare acceptability arises from the use of synonymous terms describing acceptability^1^,^4^, ^15^. Therefore, we call for a stricter designation of terms used to describe acceptability. We recommend the term 'dimension’ to define the highest or macro level describing acceptability. We also suggest the term ‘construct’ to describe the medium or meso level explaining the specific constructs of acceptability. Finally, we advocate the use of the term ‘component’ to label the unitary or micro level relating to individual information or variables explaining acceptability.

### Definition of acceptability

We analysed multiple definitions of acceptability in the literature and they all appeared to describe different aspects within the continuum of acceptability (component, construct or dimension)^5^, ^6^ with no clear-cut definition^1^,^6^. Theories used to define acceptability often drew from different disciplines especially social sciences, health psychology, health economics and public health^4^–^6^. When carefully analysed, those theories are complementary and explain the heterogeneous nature of a complex concept such as acceptability. We suggest that one way of better defining acceptability of healthcare is to consider the actor( s) who has the largest vested interest in a particular aspect of acceptability of healthcare.

Many authors have agreed that one of the best ways of approaching acceptability is from patients, healthcare providers or healthcare system managers or policy makers' perspectives^1^, ^2^, ^19^. Stakeholder analysis and actor-network theories would be one of the best ways to answer the question: Acceptability to whom?

Building on existing literature and having explored the context as well as the basic theories helping to unpack the complexity and semantic domains of acceptability, we proposed a more practical and comprehensive definition of acceptability. We defined acceptability as ‘a multi-construct concept describing nonlinear cumulative combination in parts or in whole of expected and experienced degree of healthcare from patient, provider or healthcare systems and policy perspectives in a given context.'

### Conceptual framework of acceptability

The articles in this analysis did not offer a shared framework of acceptability^1^, ^2^, ^5^. Though acceptability is widely believed to reflect patient, provider and healthcare systems or policy views^2^, ^19^, almost all frameworks have approached acceptability of healthcare from the patients perspective^1^, ^2^, ^5^ with little attention to other actors involved in networks explaining acceptability. We recommended a conceptual framework of acceptibility based on our proposed definition.

The suggested approach to our conceptual framework is based on five essential features: (1) context, (2) basic theories, (3) dependent variables, (4) independent variables and (5) applications of acceptability in public health. Context of acceptability consists of the setting, population, content, scope and focus. The basic theories that can be used to generate a shared understanding of acceptability include demand-supply sides, best-fit, mutual exclusivity, complex phenomenon, stakeholder analysis and actor-network. It should be noted that the full description of these theories does not fall within the scope of this article.

Dependent variables include a set of components that define acceptability of healthcare. The focus of acceptability either from patient, provider or healthcare system manager or policy maker viewpoints should guide the selection of relevant components. At the level of dependent variables, the researchers can only conduct descriptive analysis^7^. Privacy, attitude of healthcare providers or cleanliness of the facility are examples of dependent variables of healthcare acceptability.

Independent variables include factors that are not part of the definition of acceptability but can or have proved to have significant impact on it either positively or negatively7. Independent variables consist of predictor variables associated with acceptability of healthcare^7^. Therefore, it is possible to perform inferential statistical analysis at the level of independent variables^7^ and this is not possible at the level of dependent variables^7^. Examples of independent variables of acceptability includes factors such as age, education level and socio-economic status to name few.

With regard to applications of acceptability in public health, we designed a flexible and adaptable framework to various contexts. The essential added value of this framework is to clarify the description of acceptability of healthcare from the perspectives of the patient, provider or healthcare system manager and policy maker. In addition, the proposed framework clarifies the notion of dependent and independent variables that can guide and help wih the current confusion in literature. Furthermore, this framework provides practical and targeted application for assessing acceptability from component (micro) or unitary, construct (meso) or multi-component or dimension (macro) or multi-construct levels.

## Discussion

In this paper, we presented a coherent definition of healthcare acceptability, which we converted into a conceptual framework. We considered acceptability within the context of access and, as a multi-construct, complex concept. Our analysis confirmed that imprecise definitions of acceptability and lack of a coherent conceptual framework have hindered the application of healthcare acceptability in healthcare systems and policy^1^–^3^, ^5^, ^21^.

Our findings agreed with other publications describing acceptability as a dimension of access to healthcare^15^, ^16^,^20^. This could help in resolving some misunderstandings surrounding the definition of acceptability. Ignoring acceptability as a facet of access to healthcare would probably result in using some components that are better suited to describing other dimensions of access. This has been noted in Sekhon and colleagues' theoretical framework of acceptability (TFA) considering “Opportunity Costs” among the seven constructs of acceptability^2^. “Opportunity Cost” was defined as “the extent to which benefits, profits, or values must be given up to engage in the intervention” 2. One could argue that the construct of “Opportunity Cost” would be best-fit into the dimension of affordability also called financial access4. Nevertheless, the authors of this article understood that the “opportunity cost” could also be other than financial.

The findings from this analysis supported the claim of acceptability of healthcare as a multi-construct concept^1^,^2^, ^5^ even though not all articles agreed on the number and types of acceptability constructs^1^, ^2^, ^5^. We proposed the definition of acceptability to retain the constructs or elements of acceptability suggested by Gilson^3^ and later confirmed by other scholars^1^, ^4^, ^15^. These constructs offer a holistic explanation of acceptability, and include patient-provider, patient-healthcare and patient-community interactions. Those constructs are also called provider acceptability, healthcare acceptability and community acceptability respectively. Most articles analysed here only described specific aspects of acceptability such as relationships between patient health providers or participant and intervention2 and therefore missing some key aspects of acceptability such as the community component^2^, ^5^.

This paper was aligned with the description of acceptability as a multi-level complex concept5. Usually there are too little data describing the levels of complexity or acceptability leading to inconsistent definitions. This article added to existing literature in describing the semantic domains of acceptability corresponding to their level of complexity. The semantic domains include ‘dimension' corresponding to the highest or macro level of acceptability, 'construct’ corresponding to medium or meso level of acceptability and ‘component’ corresponding the lowest or micro level of acceptability. The results obtained concur with previous publication advocating for the mutual exclusivity of the healthcare acceptability constructs where no component is categorised under more than one construct^2^, ^5^.

Our findings agreed with other studies which declared a lack of clear-cut definition of acceptability^1^, ^5^, ^21^. However, the application of complex system theories such as mathematic modelling of complex phenomenon, stakeholder analysis and actor-networks would provide insight in defining acceptability of healthcare at macro, meso and micro level. A comprehensive definition should consider patient-provider, patient-healthcare and patient-community relationships. Accordingly, we defined acceptability of healthcare as: “A multi-construct concept describing nonlinear cumulative combination in parts or in whole of expected and experienced degree of healthcarfrom patient, provider or healthcare systems policy makers in a given context.” This definition informed the development of acceptability conceptual framework.

The results from this study confirmed the lack of shared interpretation of acceptability frameworks reported in the published literature^1^, ^2^, ^6^. Lack of common understanding of acceptability frameworks significantly hampers the use of such frameworks in healthcare systems and policy. We hope that this distinctively enunciated conceptual framework would inform unbiased assessment of acceptability and create consensus on acceptability definition and its conceptualisation among public healthcare professionals.

## Conclusion

This paper proposes a workable definition of healthcare acceptability considering perspectives from patients, healthcare providers and healthcare system managers or policymakers. Drawing on existing literature, we suggested a definition of acceptability as ‘a multi-construct concept describing nonlinear cumulative combination in parts or in whole of expected and experienced degree of healthcare from patient, provider or healthcare systems and policy perspectives in a given context.’ The paper also describes a new and comprehensive conceptual framework applicable to healthcare acceptability through quantitative, qualitative and mixed methods in public health research and practice. We recommend further studies in order to validate and widely adopt the suggested definition and conceptual framework. Nevertheless, we believe this paper provides substantial information contributing toward forging consensus on the concept of acceptability definition and its framework among public health researchers and practitioners.

## Figures and Tables

**Figure 1:**
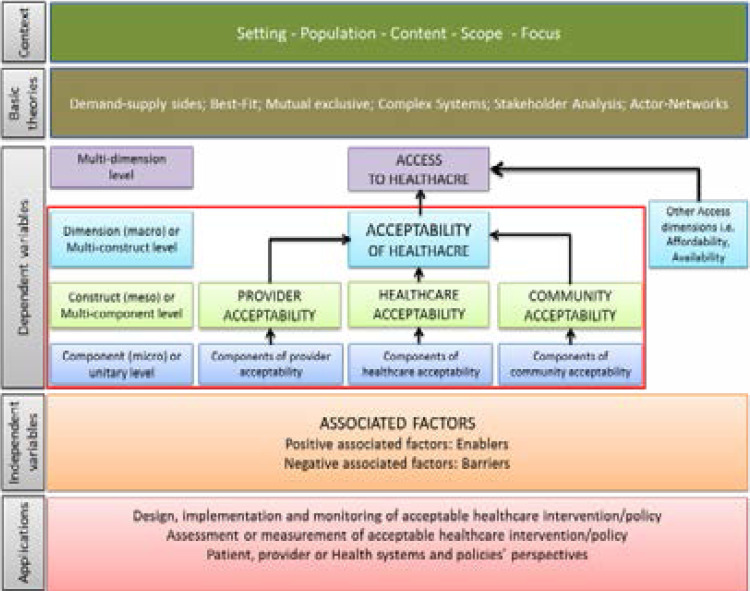
Conceptual Framework of healthcare acceptability
